# Harmonic Hopping, and Both Punctuated and Gradual Evolution of Acoustic Characters in *Selasphorus* Hummingbird Tail-Feathers

**DOI:** 10.1371/journal.pone.0093829

**Published:** 2014-04-10

**Authors:** Christopher James Clark

**Affiliations:** 1 Museum of Vertebrate Zoology, University of California, Berkeley, California, United States of America; 2 Peabody Museum of Natural History, Yale University, New Haven, Connecticut, United States of America; University of Akron, United States of America

## Abstract

Models of character evolution often assume a single mode of evolutionary change, such as continuous, or discrete. Here I provide an example in which a character exhibits both types of change. Hummingbirds in the genus *Selasphorus* produce sound with fluttering tail-feathers during courtship. The ancestral character state within *Selasphorus* is production of sound with an inner tail-feather, R2, in which the sound usually evolves gradually. Calliope and Allen's Hummingbirds have evolved autapomorphic acoustic mechanisms that involve feather-feather interactions. I develop a source-filter model of these interactions. The ‘source’ comprises feather(s) that are both necessary and sufficient for sound production, and are aerodynamically coupled to neighboring feathers, which act as filters. Filters are unnecessary or insufficient for sound production, but may evolve to become sources. Allen's Hummingbird has evolved to produce sound with two sources, one with feather R3, another frequency-modulated sound with R4, and their interaction frequencies. Allen's R2 retains the ancestral character state, a ∼1 kHz “ghost” fundamental frequency masked by R3, which is revealed when R3 is experimentally removed. In the ancestor to Allen's Hummingbird, the dominant frequency has ‘hopped’ to the second harmonic without passing through intermediate frequencies. This demonstrates that although the fundamental frequency of a communication sound may usually evolve gradually, occasional jumps from one character state to another can occur in a discrete fashion. Accordingly, mapping acoustic characters on a phylogeny may produce misleading results if the physical mechanism of production is not known.

## Introduction

Tonal sound is produced by acoustic systems via the excitation of a resonance frequency of a structure, such as a guitar string, vocal fold, the wing of a cricket or vane of a feather [1]–[4]. It is easy to imagine how, over evolutionary time, a communication sound might gradually change through small, gradual changes in the resonant structure. For instance, a small increase in stiffness slightly increases frequency, similar to the effect of tightening a guitar string, and the acoustic frequency evolves gradually. However, in addition to this gradualism hypothesis, there is another possibility, in which sounds evolve in a ‘punctuated’ fashion. All resonant structures contain multiple resonance frequencies (harmonics), and changes in excitation of the structure can cause the dominance of one of these other frequencies rather than the original. For example, that same guitar string has pinch harmonics, which become dominant if the plucked guitar string is pinched on a node. Over evolutionary time, the dominant frequency of a sound could hop from one harmonic to another, without passing through intermediate frequencies, a sort of ‘punctuated’ evolution [5] of an otherwise continuous character. Kingston and Rossiter [6] called this ‘harmonic-hopping’ and argued that this explained differences in echolocation frequency in morphs of a horseshoe bat. Robillard et al. [5] demonstrated a similar hop in the evolution of cricket stridulation, and showed that the ancestral structural resonance frequency remained present in the cricket wing, after the hop to a much higher acoustic frequency. They termed this latent frequency a ‘ghost’ frequency, as in ‘the ghost of phenotypes past’. Here, I provide another example of both harmonic hopping and a ghost frequency, in the course of exploring how two unique, autapomorphic mechanisms of sound production arose in the tails of *Selasphorus* hummingbirds. Determination of the mechanistic details of how a phenotypic character is produced allows a nuanced description of how it evolves, and in this case, allows rejection of simple models of character evolution.

Many birds produce non-vocal sounds, termed ‘sonations’ when intentionally produced during a display [7]–[10]. Perhaps the single largest radiation of sonating birds are the ∼38 species in the ‘bee’ hummingbird clade: males produce sounds with their tail-feathers during high-speed courtship dive displays [11]–[13]. Each species has unique tail morphology which it uses to produce unique sounds, suggesting this sexual character has rapidly diversified under sexual selection.

As sound production is not the primary function of most bird feathers, the relationship between feathers with seemingly modified shape and the sounds they putatively produce is seldom clear from morphology alone. Experimental evidence can clarify the relationship(s). Experimental manipulation of live birds allows tests of which feather is necessary for production of a particular sound, while experiments that attempt to reproduce the sound, such as eliciting sounds from feathers in a wind tunnel, establish physical sufficiency and test the mechanism of sound production [11], [12], [14]–[16]. Most experiments on how aeroelastic flutter of feathers generates sound have, for simplicity, focused on how single feathers generate sound [14], [15], [17], [18].

Most birds that sonate by fluttering feathers have multiple adjacent feathers with seemingly modified structure [19]–[21]. Wind tunnel experiments demonstrate that aerodynamic interactions between neighboring feathers can alter the sounds they produce, because fluttering feathers in close proximity can act as aerodynamically coupled oscillators [11], [22]. I posit these feather-feather interactions are widespread. This implies that a feather with seemingly modified shape may play a role in sound production through interactions with neighboring feathers, even if experiments show that feather itself is unnecessary or insufficient for sound production *per se*. Therefore, to incorporate possible feather-feather interactions with the logic of experiments that test necessity and sufficiency of individual feathers, I next organize the types of feather-feather interactions demonstrated thus far under a source-filter framework, cast explicitly in terms of necessity and sufficiency. I then demonstrate that this source-filter model makes predictions that can explain how novel mechanisms of sound production have evolved within the hummingbird genus *Selasphorus*.

### A source-filter model of feather-feather interactions

I define the sound source as the minimum set of feathers that is both necessary and sufficient to produce *quantifiable* components of the sound of interest. The simplest source is a single feather (or feather part) that is a lynchpin for sound production (Table 1). Alternately, two or more neighboring feathers together comprise the sound source. If each is individually sufficient but unnecessary, they are co-sources. Or, if individual feathers are neither necessary nor sufficient to produce the sound, whereas they are in aggregate, then they are an aggregate source. Examples of studies that have demonstrated each of these types of sources (lynchpin, co-sources, aggregate sources) are provided in Table 1.

**Table 1:** Examples of types of sound sources of feather-generated sounds.

Species	Source, type	Filter	Evidence	Citation
Anna's Hummingbird (*Calypte anna*)	R5 ‘lynchpin’	R4	R5 alone is necessary, sufficient for sound	[11], [12]
Costa's Hummingbird (*C. costae*)	R5 ‘lynchpin’	R4?	R5 is necessary, sufficient for sound	[13]
Black-chinned Hummingbird (*Archilochus alexandri*)	R5 ‘lynchpin’	R4?	R5 alone is necessary, sufficient for sound	[16]
Calliope Hummingbird (*Selasphorus calliope*)	R1, R2, R3, R4?, aggregate source	R5?	Individual feathers neither necessary, sufficient, whereas R1-R3 are when tested in aggregate	[22]
Red-billed Streamertail (*Trochilus polytmus*)	P8, P9 co-sources	P7?, P10?	P8, P9 are individually sufficient but not necessary	[38]
Common Snipe (*Gallinago gallinago*)	Outer tail-feather, lynchpin	None?	Outer tail feather is necessary, sufficient for winnowing sound	[17], [39]

I define filters as the adjacent feather(s) to which the source feather(s) are aerodynamically coupled. I posit that essentially all flight feathers are coupled to their immediate neighbors through near-field interactions [11]. Due to this coupling, these neighbors vibrate in forced response to the source, and therefore act as a filter to sound and vibration of the source. While experiments indicate these neighbors are unnecessary and insufficient to produce the sound, and therefore by definition they are not a source, they may nonetheless affect aspects of the sound that are difficult to quantify. For example, a filter may modulate amplitude by vibrating in sympathetic response to a neighboring source feather, amplifying loudness. This mechanism was demonstrated for the Anna's Hummingbird R4, which is neither sufficient nor necessary to produce quantifiable aspects of this species' dive-sound [12], and so is not a source, but in a wind tunnel, amplifies the sound generated by R5 by ∼12 dB [11]. This source-filter model predicts types of filtering not previously demonstrated, such as spectral filtering, in which a filter feather attenuates or amplifies a portion of the frequency spectrum of the source.

**Figure 1:**
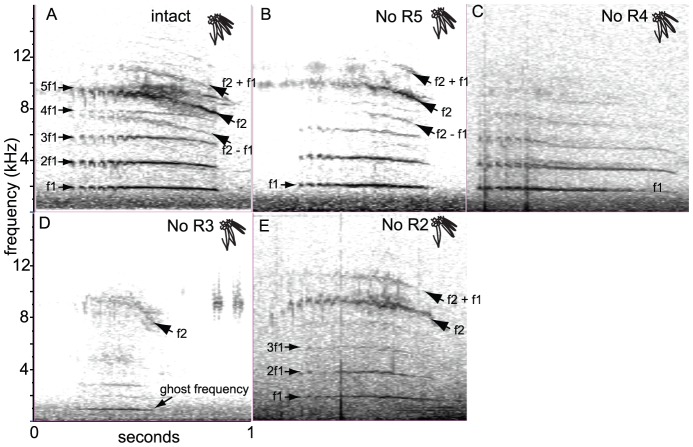
Effects of removing tail-feathers on dive sound spectrograms of Allen's Hummingbird. **A:** intact. **B-E:** after experimental removal of R2 – R5. Labeled on the left are frequencies f1, and 2^nd^ (2f1), 3^rd^ (3f1), 4^th^ (4f1), and 5^th^ (5f1) harmonics of f1 (harmonics present in **B** and **C** are not labeled). On the right is labeled f2, heterodyne interaction frequencies (f2±f1). Recorded at 48 kHz, presented with a 1024-sample FFT window. See text and Table 3 for more information.

In addition to source-filter interactions, this model also makes predictions about coupled-source interactions. For example, if two coupled sources vibrate at different frequencies, sideband (‘heterodyne’) interaction frequencies will result. Data showing this occurs in Allen's Hummingbird were briefly sketched in Clark et al. [11]. During their courtship dive males produce a shrill whining sound that includes frequencies f1 and f2 (Figure 1). Frequency f1 has a ∼1.9 kHz fundamental frequency with a stack of 5 or more integer harmonics that shows little frequency modulation through the dive. A fainter second frequency (f2) is usually apparent. Early in the dive f2 is indistinguishable from the 5^th^ harmonic of f1 at ∼ 9 kHz, but in some dives, diverges from this harmonic, descending to ∼7 kHz as the male slows through the bottom of the dive [23]. Hypothesized interaction frequencies of f2±f1 are present in good recordings (Figure 1A). If oscillators vibrating at frequencies f1 and f2 are coupled, heterodyne interaction frequencies of f2±f1 appear, as previously observed in bird syringes [24], [25]. Here, I present the full set of experiments supporting this conclusion.

I then show that, within the hummingbird genus *Selasphorus*, both Allen's Hummingbird and Calliope Hummingbird have evolved complex, autapomorphic sound production mechanisms. The source-filter model provides a hypothesis of how the novel, autapomorphic sounds of the Calliope Hummingbird and Allen's Hummingbird have evolved. In particular, in the source-filter paradigm proposed here, source and filter are not independent (unlike the source-filter model of vocalizations [26]. Therefore, any evolved change in a source feather will change its interactions with neighboring filters. As a result, any selection on acoustic properties of sounds produced by the source will also result on selection on the filters to evolve in response, since the entire system is presumably tuned. As the source feather induces flutter or vibrations in its neighbors, it may be easy for a neighbor to evolve from a filter to a component of the source, i.e. to attain necessity or sufficiency.

## Methods

### Experiments on Allen's Hummingbird

Field-work on Allen's Hummingbirds was conducted under collecting permits from the East Bay Regional Park District, California State Parks, Cal Fish & Game (permit #SC-006598), US Fish and Wildlife Service (permit MB087454-0), Patuxent Bird Banding Lab (permit #23516), and approval from the UC Berkeley Animal Care and Use Committee at UC Berkeley to R. Dudley (#R282-0310). This research was conducted in the spirit of the ethical use of wild birds in research [27], and caused minimal suffering (plucking feathers causes only momentary pain, and they regrew in approximately 5 weeks).

To determine the necessity of individual tail-feathers on the production of sounds f1 and f2, I performed manipulations on wild male Allen's Hummingbirds, in a population of *S. s. sasin* breeding at the ‘Albany Bulb’ portion of the Eastshore State Park, Albany, CA in 2005-2009 (GPS: 37.890, −122.317), and one male *S. s. sedentarius* at the Santa Cruz Island Reserve (GPS: 33.997,−119.725) in 2006. The technique was similar to the experiments in Clark and Feo [12]: focal males were sound-recorded with a shotgun microphone (Sennheiser ME67) and a 16-bit digital recorder (Marantz PMD 670, sample rate: 48 kHz) as they performed natural displays. These individuals were then captured, banded, had one or more pairs of rectrices plucked (all manipulations were bilaterally symmetrical), given a unique marking (with white-out) on the top of the head to enable field re-identification, and released. A fraction of the manipulated males were then later relocated on their territories, and their display sounds were recorded a second time, before the manipulated tail-feathers regrew.

Correctly identifying post-manipulation birds was a challenge: Males did not dive to mounts or caged birds, they were often difficult to visually follow on their territory, they often displayed on neighboring territories, and marked (i.e. manipulated) birds sometimes switched territories. Mistaken attribution of a pre-manipulation dive to the wrong individual will only rarely produce misleading results because most individuals in a population are competent. By contrast, attributing post-manipulation dives to the correct bird was essential. If a given feather is a lynchpin, crucial to production of a given sound, its experimental removal is predicted to completely eliminate the bird's ability to produce the sound. Therefore, a single observation of a post-manipulation bird producing a sound is sufficient to falsify the hypothesis that the manipulated feather produced it, assuming the bird was correctly identified. Therefore I did not use recordings in which my field notes suggested reason to suspect misidentification.

At the beginning it was not clear whether the tail produced any part of the sound, so a preliminary male had four rectrices (R2-R5) plucked to determine whether the tail was responsible for any part of the dive-sound. Based on the positive result, I then performed 15 experimental manipulations on 11 Allen's hummingbirds. I first removed pairs of rectrices from two males in order to better isolate which part of the tail generated specific sounds. One male had both R2 and R3 removed; and another, R4 and R5 removed. Based on the result, the next 13 manipulations were each of one rectrix, to test precisely which rectrices were necessary to produce individual parts of the dive-sound. Four males were re-used due to a limited number of suitable males; in each case the first manipulation had no detectable effect on the dive-sound, and the male re-grew tail feathers before the second manipulation. Three of the four individual males that underwent multiple experimental treatments first had R5 plucked, then weeks later, R4 plucked; the remaining male had R2 plucked followed later by R3.

### Other Selasphorus and outgroups

Sound recordings of natural displays of Rufous Hummingbirds (*S. rufus*) were obtained in Oregon in April 2009 (GPS: 45.73, −123.9). Display data for other outgroups were obtained from studies listed in Table 2. No data on the display of the Glow-throated Hummingbird (*S. ardens*) are available, but it appears to fall within the Scintillant-Volcano clade (McGuire pers comm) and its tail morphology is similar to its sister taxa [28] implying similar character states.

**Table 2:** Character states for *Selasphorus* hummingbirds.

Clade	Tail-feather source	mechanism	reference
Calliope	R1, R2, R3, R4, R5?	Feathers flutter and hit each other, producing buzzing sound	[22]
Allen's	R3, R4	R3 flutters via tip mode; R4 flutters via trailing vane mode	This study
Rufous	R2	R2 flutters via tip mode	This study
Broad-tailed	R2	R2 flutters via tip mode	[40]
Scintillant - Volcano	R2	R2 flutters via tip mode	[41]
*Atthis* sp	N/A	Does not dive	[42], Clark unpublished
Outgroups	R5 and/or R4	R5 and/or R4	[12], [16], Clark unpublished

### Analyses

A molecular phylogeny of the bee hummingbirds was obtained from McGuire et al. [29] and supplemented with additional taxa (McGuire pers comm). Sound recordings were analyzed in Raven 1.3 (www.birds.cornell.edu/raven). Sound recordings associated with this study have been deposited in the Museum of Vertebrate Zoology (Accession #14752).

## Results

### Allen's Hummingbird

Dive sound component f1 was produced nearly 100% of the time in unmanipulated birds, while sound f2 was distinguishable in 36 of 46 pre-manipulated dives (Table 3). In some recordings f2 was not distinguishable from the 5^th^ harmonic of f1, and f2 was faint and tended to be absent in low-quality sound recordings. In good sound recordings, additional faint, frequency-modulated sounds were detected at frequencies f1±f2 (Figure 1A). As these sounds were even fainter than f2, they were present in fewer recordings of pre-manipulated birds (24 out of 46 of dives; Table 3).

**Table 3:** Effects of removal of tail-feathers of Allen's hummingbird on fraction of dives containing dive-sound components. See also figure 1.

	N (birds)	f1		f2		f1±f2		n (dives)	
Removed feather		pre	post	pre	post	pre	post	pre	post
R2, R3	1	100%	0%	100%	100%	100%	0%	3	1
R4, R5	1	100%	100%	100%	16.6%^a^	66%	0%	3	6
R5	3	100%	100%	80%	73%	30%	60%	10	15
R4	3	100%^b^	100%	80%^b^	0%	30%^b^	0%	10^b^	9
R3	3	100%	0%^c^	59%	100%	47%	0%	17	10
R2	4	100%^d^	93%	85%^d^	80%	75%^d^	13%	20^d^	15

Note.– There were 46 total pre-manipulation dives from 11 birds; 4 individuals underwent multiple manipulations, so data for their pre-manipulation dives are repeated^b,d^. Bold numbers are statistically significant decreases (Cochran-Mantel-Haenszel test, p<0.01, bird ID as replicate).

a one post-manipulation dive contained f2, apparently due to a misidentified individual.

b control data duplicated from previous row; each bird that had R5 manipulated later had R4 manipulated, after R5 had regrown.

c sounds at ∼1 kHz frequency (the ghost frequency) produced in some dives.

d one bird had R2 and later R3 plucked; its 9 pre-manipulation dives are included in both totals.

Effects of experimental manipulation on dive-sounds of Allen's Hummingbird are presented in full in Table 3. The experimental manipulations of wild male Allen's Hummingbirds show that R5 was not necessary for any sound component, as removing R5 did not eliminate production of frequencies f1, f2, or f2±f1 (Figure 1B). Removing R4 eliminated production of f2 and f1±f2 (Figure 1C) in all but one recording, and I posit the bird in this one recording was misidentified (see methods) and so can be disregarded. Removing only R3 completely eliminated production of sound f1 and f1±f2, and a new, faint sound (the ghost frequency) with a fundamental of ∼1 kHz appeared in a few recordings (Figure 1D). R2 was not necessary for any of the sounds, as removing only R2 did not eliminate production of either f1, f2, or f1±f2 (Figure 1B), although subjectively, it did seem that dive sound loudness was reduced. To summarize, experimental manipulations of wild birds showed that R3 is necessary to produce sound f1 and R4 to produce sound f2, and both are necessary to produce the hypothesized f1±f2 interaction frequencies.

**Figure 2:**
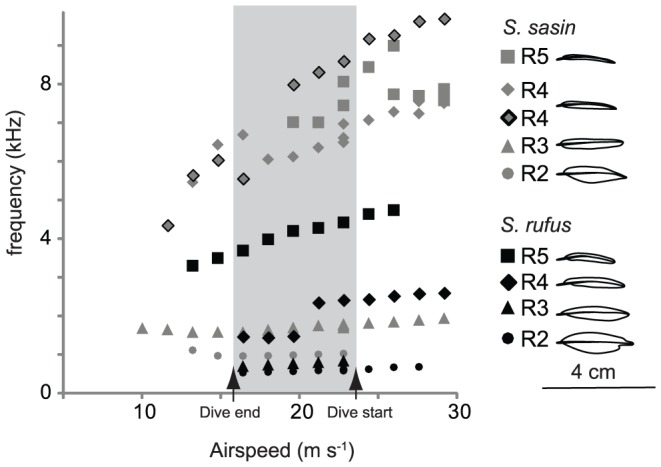
Fundamental frequency of Allen's and Rufus tail feathers as a function of airspeed. Gray bar indicates speed range of 17 – 26 m s^−1^, which corresponds to estimated dive speed for Allen's through during the dive [23]. These data show that both Allen's Hummingbird R5 and R4 are sufficient to produce sound f2, Allen's R3 is sufficient to produce sound f1, and Allen's R2 is sufficient to produce the ‘ghost frequency’ (Figure 1). Each feather, except *S. sasin* R5, was held at a constant orientation. Mode of flutter and sound frequency of some feathers varied as a function of orientation [14], which is why the two *S. sasin* R4 (gray diamonds) produced different frequencies, and is why the *S. sasin* R5 (gray squares) are not collinear (because feather orientation varied). Data reproduced from [11].

Wind tunnel experiments revealed that Allen's R4 and R5 both can produce sounds via a trailing vane mode of flutter (Fig. 2) that are sufficient to produce sound f2. At an airspeed of 22.8 m s^−1^, R4 produced sound at 7.3±0.4 kHz (n = 8 feathers), and frequency was lower at lower airspeeds, just as sound f2 decreases over the course of the dive. Allen's R3 fluttered via a tip mode at 1.9 kHz±0.14 (n = 8 feather, 22.8 m s^−1^) with little variation with orientation/airspeed, sufficient for sound f1, while R2 fluttered via a tip mode at ∼ 1.0 kHz, also with little variation with airspeed (Fig. 2).

The collective result of these wind tunnel experiments show that Allen's R5 and R4 are each sufficient to produce f2 of the dive-sound, while R3 is sufficient to produce sound f1. R2 is sufficient to produce the ∼1 khz sound produced by birds missing R3 (Figure 1D). Finally, R4 and R3 in close proximity are sufficient to produce the f2±f1 heterodyne interaction frequencies [11]. The combined lab and field experiments indicate that R4 and R3 alone are the sources of sounds f2 and f1, respectively, as they are both necessary and sufficient to produce them, including the f2±f1 heterodyne frequencies. Regarding R5 and R2, the wind tunnel experiments suggest they are sufficient to produce f1 and f2 of the dive sound (R2 at its even harmonics only), but the field experiments suggest they are not necessary, and therefore they are filters.

### Characters ancestral to Selasphorus

**Figure 3:**
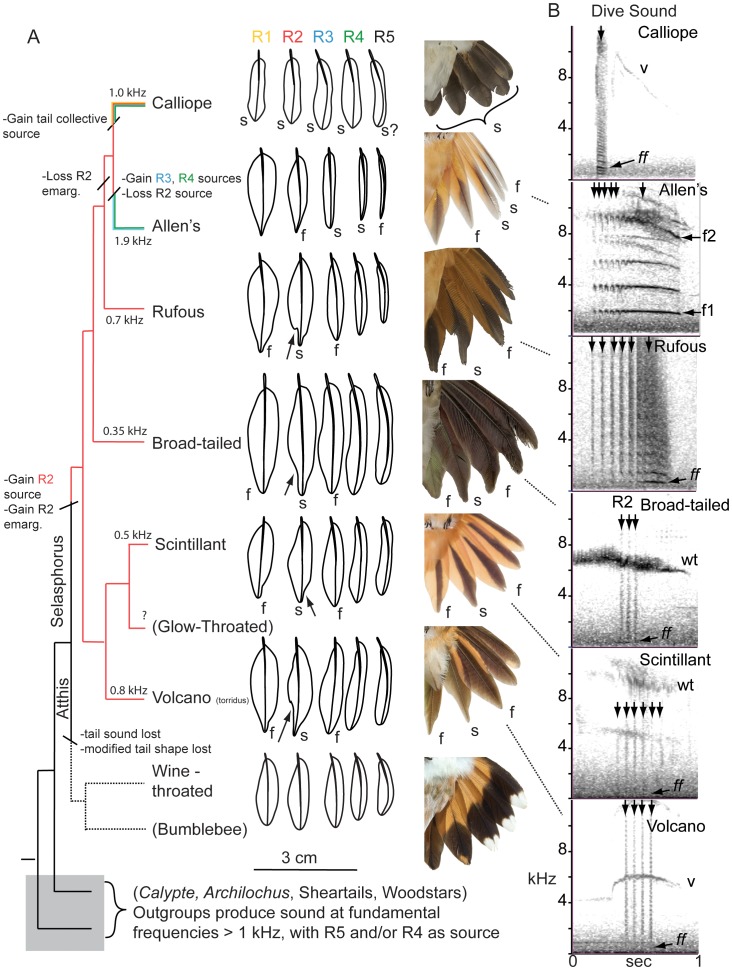
Evolution of tail morphology and dive sounds in *Selasphorus*. **A**. Phylogenetic reconstruction of emargination of R2 and which feather(s) are the source, *s*, of the dive sound. Neighboring feathers, *f*, are hypothesized to be filters. Branch colors indicate which tail-feathers are sound sources; dashed line indicates taxa that do not dive or produce any sound. R2 has an emarginated in shape (arrow) in the taxa in which it is a source. Fundamental frequency (in kHz) indicated for terminal taxa. Feather drawings are to-scale; photos are not. **B**: Spectrograms of the dive sound of male *Selasphorus* Hummingbirds (Hann, 1024-sample FFT window). Fundamental frequency of flutter indicated by *ff* and arrow. Vertical arrows indicate pulses of sound produced by individual tail-spreads. Vocalizations (*v*) or wing trills (*wt*) are also produced during the dive. Photos courtesy Anand Varma; Phylogeny from [29] and McGuire pers comm.

**Figure 4:**
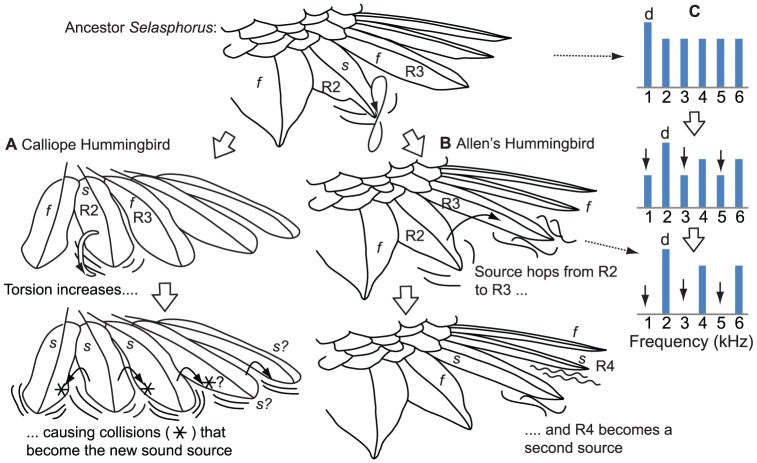
Changes in sound production mechanism from hypothesized *Selasphorus* ancestor to Calliope Hummingbird and Allen's Hummingbird. Right half of the tail is shown. Sound sources labeled *s*, filters *f*, and rectrices 2 and 3 are R2 and R3 respectively. The changes are represented in two steps, whereas the underlying trait evolution was likely continuous. C: proposed switch from a ∼1 kHz dominant frequency produced by R2 in the ancestor, to a ∼2 kHz dominant frequency produced by R3 in Allen's Hummingbird. Initially R2 is the source (top), then R3 acts as a spectral filter, amplifying the even harmonics (middle), then R3 becomes the source and the odd harmonics (black arrows) are lost (bottom). The result is the dominant frequency (d) ‘hops’ from 1 kHz to 2 kHz. See text and Figure 3 for more information.

Multiple outgroups of the *Selasphorus*-*Atthis* clade dive and produce sound with outer tail-feathers, suggest that diving and producing sound with the tail is ancestral to the bee hummingbird clade (Fig. 3). Within *Selasphorus-Atthis*, three clades (Rufous, Broad-tailed, and the Scintillant-Volcano clade) all have the same character states: R2 is the sound source, flutters with a fundamental frequency <1.0 kHz, and is emarginated (arrows in Fig. 3A); Table 1. The tip mode of flutter of R2 incorporates both transverse (bending) and torsional (twisting) components of motion, which is depicted in Figure 4A as a figure-eight trajectory of the feather's tip. Its neighbors, R1 and R3, are hypothetical filters. Given that three outgroups have these characters, the most parsimonious reconstruction is these characters have evolved in tandem on the branch leading to *Selasphorus*.

There are other plausible phylogenetic topologies apart from the one presented in figure 3, such as Allen's sister to Rufous [28], [30] or *Atthis* inside *Selasphorus* [31]. The most parsimonious ancestral character state is not different under these alternatives. From these ancestral character states, Calliope and Allen's have each evolved their unique mechanisms of sound production. Each appears to have involved separate processes that produce large mechanistic differences, based on small, continuous changes in feather shape.

### Evolution of the Calliope Hummingbird's sonation

Calliope Hummingbirds have two autapomorphic components of their sound production mechanism: the feathers exhibit a predominantly torsional mode of flutter [11], [14], and all of the tail-feathers together constitute the sound source, because the mechanism appears to be that multiple neighboring tail-feathers strike each other during each flutter cycle [22]. Given the data presented above, and the source-filter model of feather-feather interactions, the origin of each autapomorphic component has a simple explanation. The ‘tip’ modes of flutter of Calliope tail-feathers and the other *Selasphorus* lie on a continuum, with a purely bending mode at one extreme, and a purely torsional mode at the other (Figure 4). I hypothesize that small changes in feather shape in the ancestor of Calliope shifted the mode of flutter along this continuum towards torsion, resulting in the transition from the ‘tip’ modes of basal *Selasphorus* to the ‘torsional’ mode of *S. calliope* (Figure 4A).

The transition from the ancestral character state of R2 as sole source to the entire tail as a source is straightforward: as R2 evolved to a torsional mode of vibration, it began colliding with its neighbors R1 and R3, incorporating them into the source (Figure 4A). This process then may have repeated with R4 (Figure 4A); R4 and R5 do appear to be components of the source [22], though details of the mechanism are not entirely clear, so the presence of feather-feather collisions is inconclusive in the case of R4 and unlikely for R5 (as inferred from the data in Table 1 of ref 22). The sound that is now produced by Calliope, the *sputter*, is the result of physical collisions rather than flutter *per se*.

### Evolution of the Allen's Hummingbird's sonation

The evolution of the dual-source (R3 and R4) mechanism of the Allen's Hummingbird involves two changes: a switch from an R2 source to an R3 source, and the origination of an R4 source (Figs. 3, 4). The ancestral character state was an R2 source that vibrated at <1 kHz, with R3 hypothetically a filter of R2 (Figure 4). The switch to an R3 source involved changes in two character states: R3 switched from filter to source, and the frequency of vibration changed from R2′s fundamental frequency of <1 kHz to an R3 fundamental of ∼2 kHz (Figure 4B).

The gradualism hypothesis proposes a simple, intuitive, wrong explanation for how this occurred. Under the gradualism hypothesis, the change occurred because the fundamental frequency of the combined vibration gradually increased from <1 kHz to ∼2 kHz, as the source feather gradually changed from R2 to R3. Therefore, the gradualism hypothesis specifically predicts that R2's fundamental frequency is now ∼2 kHz, having gradually increased along with the gradual changes in feather shape that have taken place. This is unsupported. The wind tunnel data show that R2 has maintained the ancestral character state of a fundamental frequency of vibration of ∼1 kHz (gray circles in Figure 2), and manipulated Allen's missing R3 produce a new ∼1 kHz sound, presumably with an R2 that is free to flutter when R3 is absent (Figure 1D). These results indicate that the ancestral character state of an R2 with a ∼1 kHz mode of flutter is still present, latent, as a ghost frequency in male Allen's Hummingbird. Frequency of flutter of R2 cannot have gradually changed from the ancestral character state, because it has not changed at all (Figure 2). These data instead indicate that a new ∼2 kHz mode of vibration of R3 has evolved that now masks R2's intrinsic 1 kHz mode of vibration in Allen's Hummingbird.

The proposed source-filter model suggests a simple explanation of how this happened: R3 was initially a spectral filter of R2 (Figure 4C). Specifically, in Allen's Hummingbird, R3 presently vibrates at nearly 2 kHz plus integer multiples (4, 6, 8…), which are the even harmonics of an R2 that vibrates at 1 kHz plus integer multiples (2, 3, 4…). I propose this harmonic match is not coincidental, but rather, occurs because R3 was initially a filter of R2. As feather shapes changed, it evolved to become a spectral filter of R2, responding more strongly to and amplifying the even harmonics of R2 (Figure 4C). Feather shapes evolved further, causing the source to shift from R2 to R3. As this shift occurred, the even harmonics amplified by R3 persisted while the 1 kHz fundamental and other odd harmonics diminished and disappeared (Figure 4C). At this point the former 2^nd^ harmonic of R2 had become the new fundamental frequency of R3. Under this hypothesis, the underlying changes in feather morphology were gradual, as was the shift in sound source from R2 to R3, but the fundamental frequency of vibration hopped from 1 kHz to 2 kHz without passing through intermediate frequencies, leaving R2 with the ancestral character state of a ∼1 kHz mode of vibration, now masked by the presence of R3.

A similar process explains the origination of a second sound source in R4, which early in the dive vibrates at the 5^th^ harmonic of R3 (and in some dives is not distinguishable from the 5^th^ harmonic). I hypothesize R4 was initially also a spectral filter, responding to the 5^th^ harmonic of R3 (or, the 10^th^ harmonic of R2), then changed in shape and became a source independent of R3. The sound produced by R4 (f2) is also now frequency-modulated by airspeed to a greater degree than R3 (Figure 2), meaning that it can change in pitch to a greater degree over the course of the dive. This causes it to match the 5^th^ harmonic of R4 at some airspeeds but not others, resulting in the heterodyne interaction frequencies observed (Figure 1).

## Discussion

Phylogenetic reconstruction and a source-filter model of feather-feather aerodynamic interactions together show how two hummingbirds, the Calliope Hummingbird and Allen's Hummingbird, have each evolved unique mechanisms of sound production of their tail-feathers. In each, the autapomorphic properties of the sounds produced are the product of small changes in feather morphology, which have lead to larger changes in feather-feather interactions. The Calliope Hummingbird's sound is acoustically distinct from the sound produced by other *Selasphorus* hummingbirds, because its sound is now produced by collisions between neighboring feathers rather than the ancestral character state of flutter itself generating the sound. The Allen's Hummingbird, in turn, has switched which tail-feathers are sound sources, from an ancestor that produced a single tone with the tail-feather R2, to now produce two sounds, f1 and f2, with feathers R3 and R4 respectively. At some airspeeds the f2 and f1 are harmonically unrelated, and heterodyne interaction frequencies (f2±f1) appear, showing that these neighboring feathers act as coupled oscillators (Figure 1) [11].

Acoustic characters are sometimes mapped on a phylogeny [25], [32], [33], as any other phenotypic character. The results shown here (Figures 3, 4) suggest that fundamental frequency of an acoustic signal should be mapped with caution. In Allen's Hummingbird, the ancestral character state of producing sound with R2 at ∼1 kHz has remained latent in the phenotype as a “ghost frequency”, similar to the pattern demonstrated by Robillard et al. [5] for cricket stridulation. The dominant frequency of sound production has hopped to a harmonic without passing through intermediate frequencies (Figure 4C), exhibiting ‘punctuated’ rather than gradual change on one branch, similar to results previously shown for evolution of tonal sounds produced by bat vocalizations and cricket stridulation [5], [6]. Most or all acoustic systems characterized by tones with significant harmonics are driven by the excitation of a resonator, whether a feather, wing, or vocal fold [1], [4], [34]. Therefore, the potential for harmonic-hopping may be widespread in the evolution of acoustic systems of animals, creating potential for punctuated as well as gradual evolution of this type of acoustic character [5]. This does not imply that the underlying morphological or genetic changes were punctuated; rather, the fundamental or dominant frequency of an acoustic signal is an emergent property, subject to nonlinearities (such as thresholds) in the underlying mechanism of production. Simple models of character evolution assume a single mode of evolutionary change, such as continuous or discrete, while threshold models that combine the two are just being developed [35].

Clark et al. [11] demonstrated that fluttering feathers are oscillators, and provided two empirical examples in which fluttering feathers interact with each other, exhibiting the dynamics of coupled oscillators. The coupling demonstrated was aerodynamic (not structural), as the feathers did not touch during the experiments, but is not yet precisely understood [11]. Here I have extended these empirical results with a verbal source-filter model that treats feathers as either sources, if they are both necessary and sufficient to produce a particular sound, or filters, if they are unnecessary or insufficient, but are nonetheless likely to be coupled to a source feather. Due to aerodynamic coupling, filters vibrate in forced response to sources, and evolve in shape in response to evolved changes in the source. I propose both of these features pre-adapt them to become sources.

The source-filter model proposed here provides a conceptual framework for understanding how multiple feathers interact to produce complex sounds. The wings and tails of birds are arrays of feathers, meaning that feather-feather interactions between neighbors are potentially widespread. As most birds that produce sonations during flight have multiple adjacent modified feathers, such as red cotingas [20]; twist-wings [21]; guans [36], Little Bustard [37] or Crested Pigeon [19], feather-feather interactions are likely important for the origin and filtering of nonvocal sounds in many birds. This source-filter model therefore provides a framework for further studies of the mechanism of sound production by fluttering feathers.

There is an important distinction between my source-filter model and the classic source-filter model of vocalizations [26]. In my model, the source and filter are not partially independent as they are in vocalizations; they are coupled. I do not know of any evidence that birds have independent behavioral control over the filter feather in the same way that a human controls phonation through the independent actions of the larynx and mouth. As a result, the filter simply acts as an intrinsic component of the system, modifying the form of the produced sound. In this sense, an alternative perspective to that presented here would be to consider all feathers that could possibly play any role in the sound production as a part of the source, even though experiments show some individual feathers are not, on their own, necessary or sufficient for sound production.

Finally, this raises the issue of what is meant by “for sound production”: sounds have several physical qualities, some of which are easier to quantify than others, e.g. frequency is easier to measure than loudness. From an experimental perspective, the filter will usually be hypothetical, because, by definition, experiments will demonstrate it is unnecessary and/or insufficient for components of sound that are easy to quantify. Filters therefore lack one of the lines of evidence necessary to assign causality. For example, Allen's missing R2 had a dive-sound with similar spectral content to unmanipulated individuals (Figure 1E; Table 1), though subjectively, the sound may have been quieter. The loudness of a rapidly moving animal is difficult to quantify, due to uncertainty of distance and a shifting (and presumably directional) sound field, so I do not have the data to rigorously test for this possible loudness difference. Therefore, the conservative conclusion is that R2 is not necessary for the spectral content of the dive-sound. It is hypothetically a filter, amplifying the sound of R3, as this amplification mechanism has been demonstrated in wind tunnel experiments [11]. Accordingly, there will always be more uncertainty about the true role of a hypothesized filter, than there will be about source feathers.

I suggest that two criteria are necessary to invoke the existence of a filter: 1. the mechanism is physically plausible, for example it has been empirically demonstrated; 2. The geometric arrangement of the feathers (or similar) makes coupling likely. For example, in my arguments above, I have invoked filters under the logic that neighboring flight feathers are coupled aerodynamically. The nature of this aerodynamic coupling is not entirely clear—in the wind tunnel, it is easy to elicit an aerodynamic responses in feathers separated by a couple mm, and I occasionally elicited responses at further distances of ∼1 cm. These results came from an experimental setup not well suited to carefully map the proximity required to produce coupled-feather aerodynamic interactions. It therefore remains unknown how such aerodynamic interactions scale with size, i.e. how they may manifest in larger birds.

## References

[1] Fletcher NH (1992) Acoustic systems in biology. New York: Oxford University Press.

[2] Ewing AW (1989) Arthropod Bioacoustics neurobiology and behaviour. Ithaca, New York: Cornell University Press.

[3] Bostwick K, Elias DO, Mason AC, Montealegre-Z F (2009) Resonating feathers produce courtship song. Proceedings of the Royal Society Biological Sciences Series B 227: 835–841. 10.1098/rspb.2009.1576 PMC2842718 19906670

[4] Bennet-Clark HC (1999) Resonators in insect sound production: how insects produce loud pure-tone songs. Journal of Experimental Biology 202: 3347–3357. 10562517 10.1242/jeb.202.23.3347

[5] Robillard T, Montealegre-Z F, desutter-Grandcolas L, Grandcolas P, Robert D (2013) Mechanisms of high-frequency song generation in brachypterous crickets and the role of ghost frequencies. Journal of Experimental Biology 216: 2001–2011. 23430987 10.1242/jeb.083964

[6] Kingston T, Rossiter SJ (2004) Harmonic-hopping in Wallacea's bats. Nature 429: 654–657. 15190351 10.1038/nature02487

[7] Bostwick KS, Prum RO (2003) High-speed video analysis of wing-snapping in two manakin clades (Pipridae: Aves). The Journal of Experimental Biology 206: 3693–3706. 12966061 10.1242/jeb.00598

[8] Bostwick KS, Prum RO (2005) Courting bird sings with stridulating wing feathers. Science 309: 736. 16051789 10.1126/science.1111701

[9] Darwin C (1871) The descent of man, and selection in relation to sex. Princeton, NJ: Princeton University Press.

[10] Prum RO (1998) Sexual selection and the evolution of mechanical sound production in manakins (Aves: pipridae). Animal Behaviour 55: 977–994. 9632483 10.1006/anbe.1997.0647

[11] Clark CJ, Elias DO, Prum RO (2011) Aeroelastic flutter produces hummingbird feather songs. Science 333: 1430–1433. 21903810 10.1126/science.1205222

[12] Clark CJ, Feo TJ (2008) The Anna's Hummingbird chirps with its tail: a new mechanism of sonation in birds. Proceedings of the Royal Society of London B 275: 955–962. 10.1098/rspb.2007.1619 PMC2599939 18230592

[13] Clark CJ, Feo TJ (2010) Why do *Calypte* hummingbirds “sing” with both their tail and their syrinx? An apparent example of sexual sensory bias. American Naturalist 175: 27–37. 10.1086/648560 19916787

[14] Clark CJ, Elias DO, Girard MB, Prum RO (2013) Structural resonance and mode of flutter of hummingbird tail feathers. Journal of Experimental Biology 216: 3404–3413. 23737565 10.1242/jeb.085993

[15] Clark CJ, Elias DO, Prum RO (2013) Hummingbird feather sounds are produced by aeroelastic flutter, not vortex-induced vibration. Journal of Experimental Biology 216: 3395–3403. 23737562 10.1242/jeb.080317

[16] Feo TJ, Clark CJ (2010) The displays and sonations of the Black-chinned Hummingbird (Trochilidae: *Archilochus alexandri*). Auk 127: 787–796.

[17] Reddig vE (1978) Der ausdrucksflug der Bekassine (*Capella gallinago gallinago*). Journal für Ornithologie 119: 357–387.

[18] Bahr PH (1907) On the “Bleating” or “Drumming” of the Snipe (*Gallinago coelestis*). Proceedings of the Zoological Society of London Part 1: 12–35.

[19] Hingee M, Magrath RD (2009) Flights of fear: a mechanical wing whistle sounds the alarm in a flocking bird. Proceedings of the Royal Society Biological Sciences Series B 276: 4173–4179. 10.1098/rspb.2009.1110 PMC2821341 19726481

[20] Trail PW, Donahue P (1991) Notes on the behavior and evology of the red-cotingas (Cotingidae: *Phoenicircus*). Wilson Bulletin 103: 539–551.

[21] Lane DF, Servat GP, Valqui H T, Lambert FR (2007) A distinctive new species of tyrant flycatcher (passeriformes: tyrannidae: *Cnipodectes*) from southeastern Peru. The Auk 124: 762–772.

[22] Clark CJ (2011) Wing, tail, and vocal contributions to the complex signals of a courting Calliope Hummingbird. Current Zoology 57: 187–196.

[23] Pearson OP (1960) Speed of the Allen Hummingbird while diving. Condor: 403.

[24] Nowicki S, Capranica RR (1986) Bilateral syringeal coupling during phonation of a songbird. Journal of Neuroscience 6: 3595–3610. 3794791 10.1523/JNEUROSCI.06-12-03595.1986 PMC6568646

[25] ten Cate C (2004) Birdsong and evolution. In: Marler P, Slabbekoorn H, editors. Nature's Music: the Science of Birdsong. San Diego, CA: Elsevier Academic Press.

[26] Titze IR (2008) Nonlinear source-filter coupling in phonation: theory. Journal of the Acoustical Society of America 123: 2733–2749. 18529191 10.1121/1.2832337 PMC2811547

[27] Gaunt AS, Oring LW, Able KP, Anderson DW, Baptista LF, et al.. (1999) Guidelines to the use of wild birds in research. Washington DC: The Ornithological Council. 1–59 p.

[28] Stiles FG (1983) Systematics of the southern form of *Selasphorus* (Trochilidae). The Auk 100: 311–325.

[29] McGuire J, Witt CC, Altshuler DL, Remsen JV (2007) Phylogenetic systematics and biogeography of hummingbirds: Bayesian and maximum likelihood analyses of partitioned data and selection of an appropriate partitioning strategy. Systematic Biology 56: 837–856. 17934998 10.1080/10635150701656360

[30] Clark CJ, Mitchell DE (2013) Allen's Hummingbird (*Selasphorus sasin*). In: Poole A, editor. Birds of North America Online. Ithaca, NY: Cornell Lab of Ornithology. pp. http://bna.birds.cornell.edu/bna/species/501.

[31] McGuire JA, Witt CC, Remsen JV, Dudley R, Altshuler DL (2009) A higher-level taxonomy for hummingbirds. Journal of Ornithology 150: 155–165.

[32] de Kort S, ten Cate C (2004) Repeated decrease in vocal repertoire size in *Streptoptelia* doves. Animal Behaviour 67: 549–557.

[33] Price JJ, Lanyon SM (2002) Resonstructing the evolution of complex bird song in the oropendolas. Evolution 56: 1514–1529. 12206250 10.1554/0014-3820(2002)056[1514:RTEOCB]2.0.CO;2

[34] Suthers RA (2004) How birds sing and why it matters. In: Marler P, Slabbekoorn H, editors. Nature's Music. San Diego, CA: Elsevier Academic Press.

[35] Felsenstein J (2012) A comparative meothod for both discrete and contiuous characters using the threshold model. American Naturalist 179: 145–156. 10.1086/663681 22218305

[36] Delacour J, Amadon D (1973) Curassows and Related Birds. New York: The American Museum of Natural History.

[37] Peterson RT, Mountfort G, Hollum PAD (2001) A field guide to the birds of Britain and Europe. New York: Houghton Mifflin Company.

[38] Clark CJ (2008) Fluttering wing feathers produce the flight sounds of male streamertail hummingbirds. Biology Letters 4: 341–344. 18505711 10.1098/rsbl.2008.0252 PMC2610162

[39] Tuck L (1972) The snipes: a study of the genus Capella. Ottawa: Canadian Wildlife Service.

[40] Clark CJ, Feo TJ, Bryan KB (2012) Courtship displays and sonations of a male Broad-tailed × Black-chinned Hummingbird hybrid. Condor 114: 329–340.

[41] Clark CJ, Feo TJ, Escalante I (2011) Courtship displays and natural history of the Scintillant (*Selasphorus scintilla*) and Volcano (*S. flammula*) hummingbirds. Wilson Journal of Ornithology 123: 218–228.

[42] Zyskowski K, Peterson AT, Kluza DA (1998) Courtship behaviour, vocalizations, and species limits in *Atthis* hummingbirds. Bulletin of the British Ornithologists Club 118: 82–90.

